# Supplementary material for: Neuromuscular Response during Different Side-Cutting Maneuvers and Its Influence on the Risk of Knee Injuries
Source: Sports (Basel). 2023 Oct 3;11(10):190. doi: 10.3390/sports11100190 (PMC10611348; doi:10.3390/sports11100190)
Supplement: Supplementary file 1 [file sports-11-00190-s001.zip › sports-2572197-supplementary.pdf]

Supplement A

Description of each training program´s exercise

Week	*Minutes*	*Sport Actions*	*Sports Equipment*	*Total Exercises*	*Series-rest*	*Rep*	*Observations*
1	40´	Whole Body Vibration Exercise (30Hz-4mm)	Vibratory platform	5	1-1´	10	
		Proprioceptive Training	Balance platform	5	1-1´	10	
		Eccentric Training	Russian belt	5	1-1´	10	No load
		Suspension Training	TRX®	4	1-1´	10	
		Proprioceptive Training + balance	Fitball	4	1-1´	10	
2	45´-50´	Whole Body Vibration Exercise (30Hz-4mm)	Vibratory platform	5	1-1´	20	
		Proprioceptive Training	Balance platform	5	1-1´	10	Closed eyes
		Eccentric Training	Russian belt + foam ball + disk	5	1-1´	10	External load (~10% BMI)
		Suspension Training	TRX®	4	1-1´	12	
		Proprioceptive Training + balance	Fitball	4	1-1´	10	Closed eyes
3	45´-50´	Whole Body Vibration Exercise (30Hz-4mm) + jump	Vibratory platform	4	1-1´	10	
		Proprioceptive Training + jump	Balance platform	4	1-1´	10	
		Coordination	Agility ladder	5	1-1´	2	

		Eccentric Training	Theraband	4	1-1´	10	
		Proprioceptive Training + balance	Fitball	3	1-1´	10	
4	45´-50´	Whole Body Vibration Exercise (30Hz-4mm) + jump	Vibratory platform	4	1-1´	20	
		Proprioceptive Training + jump	Balance platform	4	2-1´	10	
		Coordination	Agility ladder	5	1-1´	4	
		Eccentric Training	Theraband	4	1-1´	16	
		Proprioceptive Training + balance	Fitball	3	1-1´	16	
5	50´-55´	Whole Body Vibration Exercise (30Hz-4mm) + jump	Vibratory platform	3	1-1´	20	Closed eyes
		Proprioceptive Training + jump	Balance platform	4	1-1´	10	With turns
		Coordination Exercise	Agility ladder	5	1-1´	6	
		Eccentric Training + balance	Theraband + foam ball	4	1-1´	10	Closed eyes
		Proprioceptive Training + balance	Fitball	3	1-1´	10	Closed eyes
6	50´-55´	Whole Body Vibration Exercise (30Hz-4mm) + balance	Vibratory platform + balance platform	4	1-1´	10	

		Proprioceptive Training + cross jumping	Balance platform	3	1-1´	10	
		Isometric + balance	Russiant belt + balance platform	2	1-1´	10	
		Suspension Training + balance	TRX® + balance platform	3	1-1´	10	
		Proprioceptive Training + balance	Fitball, balance platform + foam ball	2	1-1´	10	
		Coordination + balance	Agility ladder + balance platform	3	1-1´	2	
7	50´-55´	Whole Body Vibration Exercise (30Hz-4mm) + balance	Vibratory platform + balance platform	4	1-1´	16	
		Proprioceptive Training + cross jumping	Balance platform	4	1-1´	8	Closed eyes
		Isometric + balance	Russian belt + balance platform	2	1-1´	10	External load (~10% BMI)
		Suspension training + balance	TRX® + balance platform	3	1-1´	16	
		Proprioceptive Training + balance	Fitball + balance platform + foam ball	2	1-1´	10	With foot change
		Coordination + balance	Agility ladder + balance platform	3	1-1´	4	
8	50´-55´	Proprioceptive Training + balance + jump	Agility Ladder + foam ball + balance board	4	1-1´	10	Closed eyes<br>Cross jumping
		Proprioceptive Training + balance	Cama elástica + balance platform + foam ball	6	1-1´	10	

		Balance	Fitball + balance platform + foam ball	2	1-1´	10	
		Proprioceptive Training + coordination	Agility ladder + balance platform	3	1-1´	8	
9	55´-60´	Proprioceptive Training + balance	Balance platform + balance board + foam ball	3	1-1´	10	Closed eyes
		Proprioceptive Training + balance + jump	Cama elástica + balance platform + foam ball	5	1-1´	10	
		Balance	Balance board + foam ball	2	1-1´	10	Closed eyes
		Proprioceptive Training	Fitball + foam ball	1	1-1´	10	Closed eyes and cross support
		Coordination + Proprioceptive Training + balance	Agility Ladder + balance platform + cama elástica	4	1-1´	2	
10	55´-60´	Proprioceptive Training + balance + turns	Balance platform + balance board	3	1-1´	10	Upside-down balance platform
		Proprioceptive Training + balance + jump	Cama elástica + balance platform + foam ball	5	1-1´	10	Closed eyes
		Balance	Balance board + foam ball	1	1-1´	10	Closed eyes
		Proprioceptive Training	Fitball + foam ball	1	1-1´	10	Closed eyes and cross support
		Coordination + Proprioceptive Training + balance	Agility ladder + balance platform y cama elástica	3	1-1´	4	

		Eccentric Training + Proprioceptive Training	Theraband + cama elástica	2	1-1´	10	
11	55´-60´	Proprioceptive Training + balance + turns	Balance platform + balance board	3	1-1´	16	
		Proprioceptive Training + balance + jump + turns	Cama elástica + balance platform + foam ball	6	2-1´	10	
		Balance	Balance board + foam ball	2	1-1´	10	
		Proprioceptive Training	Fitball and foam ball	1	1-1´	20	
		Coordination + Proprioceptive Training + balance	Agility ladder + balance platform y cama elástica	3	1-1´	6	
		Eccentric Training + Proprioceptive Training	Theraband + cama elástica + foam ball	2	1-1´	20	
12	55´-60´	Proprioceptive Training + balance + turns	Balance platform + balance board	4	1-1´	16	With stop tasks
		Proprioceptive Training + balance + jump + turns	Cama elástica, balance platform + foam ball	6	1-1´	16	
		Balance	Balance board + foam ball	2	1-1´	20	
		Coordination + Proprioceptive Training + balance + jump	Agility ladder + balance platform y cama elástica	3	1-1´	6	
		Eccentric Training + Proprioceptive Training	THeraband + cama elástica + foam ball	2	1-1´	16	
